# Prehospital airway management: the patient needs oxygen!

**DOI:** 10.1186/1757-7241-16-3

**Published:** 2008-07-21

**Authors:** Harald V Genzwuerker

**Affiliations:** 1 Clinic of Anesthesiology and Critical Care Medicine, University Hospital Mannheim, Germany

## Commentary

The current guidelines of the European Resuscitation Council (ERC) for advanced cardiac life support recommend that endotracheal intubation "should be attempted only if the healthcare provider is properly trained and has adequate ongoing experience with the technique." [1] One would consider anaesthesiologists to be among those who should be able to fulfill these recommendations quite easily. Interestingly, Sollid and colleagues [2] found that anaesthesia specialists and trainees who were working as helicopter emergency medical services (HEMS) physicians felt that they did not perform a sufficient number of annual intubations to maintain this important skill. An evaluation of one rural and two urban ambulance bases showed that the emergency physicians responding to prehospital calls performed one intubation every 2 to 7 months, depending on the case load of the ambulance base and the number of shifts worked by the individual physicians [3]. Therefore, I wholeheartedly agree with the conclusions reached by Sollid and colleagues that prehospital emergency physicians require improved training methods and systems to perform airway management under adverse conditions with a high probability of success.

Without adequate skills, not only in intubation but also in the verification of tracheal tube position, following advanced life support guidelines may not be possible without considerable risks for the patients. When German HEMS physicians assessed the tube position in patients who were initially intubated by other emergency physicians, the percentage of oesophageal intubations (6.7%) was unacceptably high [4]. Many of these "field airway management disasters" [5] could have been avoided by better training in intubation technique, recognition of the paramount importance of ventilating the lungs with a face mask or via a supraglottic airway device, and by the use of equipment to verify tube position, such as capnometry [6].

While the new guidelines of the Scandinavian Society for Anaesthesiologists and Intensive care medicine (SSAI) for prehospital airway management continue to recommend intubation by anaesthesiologists to secure the airway in emergencies, the importance of personal experience and skill level is pointed out as critical, in addition to the fulfillment of formal qualifications [7]. These new recommendations are a wonderful example of how to convey the basically simple, yet simultaneously complex concept of emergency airway management: The goal is to deliver as much oxygen as possible (and needed) at all times! Factors to be considered when choosing the most appropriate technique are the patient's state and anatomy, the situation at the scene, the distance to the hospital. In addition, the provider's skills and experience with various techniques to provide adequate ventilation, the equipment available and ready for operation, and any other factors that may influence the availability of oxygen on a cellular level have to be strictly observed. The authors point out in a very straightforward yet evidence-based way that for many health care providers possessing only basic to intermediate skills, the options for providing ventilation should be limited to avoid harming the intended goal of oxygen delivery. Training for these providers should focus on delivering good quality qualified performance of basic life support, including for example the lateral recovery position as the least invasive measure in patients with some degree of airway reflexes before considering other techniques. The importance of the use of supraglottic airway devices as alternatives to intubation, as well as – and possibly even more important – to the use of face mask ventilation, is pointed out in the SSAI and the ERC guidelines [7,1]. Based on the results of Sollid and colleagues [2], these recommendations should be extended to rescuers who possess advanced skills. Adequate training opportunities, programmes and requirements, as well as a restriction of the number of health care providers involved in professional rescue systems (to ensure adequate training levels), are among the strategies that are necessary for improving prehospital airway management. All efforts should focus on understanding a simple truth: emergency patients need oxygen, and they do not care how or from whom they receive it!

## Competing interests

Consulting and lecturing fees from VBM and Ambu.
